# A redox-inflammasome threshold model of ovulation: a hypothesis on NLRP3-Mediated regulation

**DOI:** 10.3389/fphar.2026.1902869

**Published:** 2026-10-13

**Authors:** Zhenghong Zhang, Defan Wang, Qinghe Lin, Pingting Guo, Zhengchao Wang

**Affiliations:** 1 Provincial Key Laboratory for Developmental Biology and Neurosciences, Key Laboratory of Optoelectronic Science and Technology for Medicine of Ministry of Education, College of Life Sciences, Fujian Normal University, Fuzhou, China; 2 Fujian Provincial Key Laboratory of Reproductive Health Research, School of Medicine, Xiamen University, Xiamen, China; 3 College of Animal Science, Fujian Agriculture and Forestry University, Fuzhou, China

**Keywords:** inflammatory threshold, mitochondrial ROS, NLRP3 inflammasome, ovarian inflammation, ovulation, redox signaling

## Abstract

Ovulation is a highly coordinated biological process involving endocrine regulation, tissue remodeling, immune activation, metabolic adaptation, and redox signaling. Although inflammatory responses are recognized as essential components of follicular rupture, the mechanisms integrating oxidative stress, metabolic alterations, and inflammasome activation during ovulatory progression remain incompletely understood. Here, we propose a hypothesis-driven redox-inflammasome threshold model of ovulatory regulation, in which NLRP3 inflammasome signaling may serve as an important integrator of redox and metabolic cues that modulate inflammatory remodeling during the periovulatory period. This model suggests that successful ovulation requires a precisely balanced inflammatory threshold, whereas insufficient or excessive inflammasome activation may disrupt follicular homeostasis and contribute to ovarian dysfunction. Importantly, NLRP3 is proposed not as an indispensable determinant of follicular rupture, but as one regulatory component within a complex network involving cytokines, prostaglandins, matrix metalloproteinases, immune cells, and hormonal signals. By integrating current evidence linking mitochondrial reactive oxygen species, metabolic stress, and inflammation, this hypothesis provides a conceptual framework for understanding ovulation as a redox-sensitive immune–metabolic process. Future studies using cell type-specific NLRP3 manipulation, quantitative redox analysis, and single-cell approaches will be required to determine the physiological contribution of inflammasome signaling to ovulatory regulation.

## 1 Introduction

Ovulation is a tightly coordinated reproductive event primarily initiated by LH surge within hypothalamic-pituitary-ovarian axis (Robker et al., 2018; Zikopoulos et al., 2026). Although traditionally considered a hormone-driven process, accumulating evidence indicates that ovulation shares remarkable similarities with acute inflammatory responses at both cellular and molecular levels, including leukocyte recruitment, inflammatory mediator production, vascular remodeling, increased vascular permeability, and ECM degradation (Duffy et al., 2019; Moustakli et al., 2026). Therefore, ovulation has increasingly been recognized as a precisely controlled sterile inflammatory process in which transient immune activation facilitates tissue remodeling required for follicular rupture.

Following the LH surge, a localized inflammatory microenvironment is rapidly established within the periovulatory follicle. Neutrophils, macrophages, and T cells are recruited to the follicular region and contribute to the release of inflammatory mediators, including IL-1β, TNF-α, IL-6, and PGE2 (Richards et al., 2002a; Tariq et al., 2026). In parallel, the expression and activation of MMPs promote degradation of collagen fibers and basement membrane components within the follicular wall, facilitating follicular rupture (Moustakli et al., 2026; Curry and Osteen, 2003). These observations indicate that ovulation requires a coordinated inflammatory remodeling program rather than solely endocrine stimulation. However, how hormonal signals are integrated with inflammatory and metabolic cues to precisely regulate the timing and extent of follicular rupture remains incompletely understood.

Recent studies have highlighted the importance of redox signaling in regulating ovulatory progression. ROS levels increase within periovulatory follicles following the LH surge, and physiological ROS production is required for processes including follicular maturation, steroidogenesis, and tissue remodeling (Shkolnik et al., 2011; Sorić et al., 2026). In particular, mitochondria-derived ROS (mtROS) are increasingly recognized not merely as metabolic byproducts but as signaling molecules capable of modulating inflammatory pathways, protease activation, and cellular responses to stress (Zhang et al., 2012; Varga et al., 2025). Meanwhile, antioxidant systems, including GSH and SOD, undergo dynamic regulation to maintain redox homeostasis during this highly active biological process (Agarwal et al., 2005; Xu and Núñez, 2023; Zhang and Wang, 2020; Shi et al., 2020). Thus, ovulation may represent a transient redox-adaptive event in which controlled oxidative signaling contributes to successful follicular remodeling, whereas excessive oxidative stress may promote pathological ovarian inflammation.

Among the molecular pathways linking cellular stress signals with inflammation, the NLRP3 inflammasome has attracted considerable attention. NLRP3 is an innate immune sensor that responds to diverse danger-associated signals, including mitochondrial dysfunction, ROS accumulation, ATP release, potassium efflux, and organelle damage (Xu and Núñez, 2023; Zhang and Wang, 2020; Shi et al., 2020). Upon activation, NLRP3 recruits the adaptor protein ASC and pro-caspase-1 to form an inflammasome complex, resulting in caspase-1 activation and maturation of IL-1β and IL-18 (Zhang and Wang, 2020; Shi et al., 2020; Fu and Wu, 2023). Importantly, IL-1β is a well-established mediator involved in periovulatory inflammatory remodeling and can regulate downstream processes such as MMP expression and ECM degradation (Takahashi et al., 2006). These findings suggest that NLRP3 signaling may represent a potential molecular connection between redox alterations and inflammatory events during ovulation.

However, whether NLRP3 directly regulates physiological follicular rupture remains unresolved. Existing studies provide evidence that inflammasome-related pathways participate in ovarian inflammation and reproductive disorders, but the precise physiological contribution of NLRP3 during normal ovulation has not been fully established. In addition, genetic studies showing improved ovarian function following NLRP3 deficiency in aging models suggest that the biological effects of NLRP3 are context-dependent and may differ between physiological inflammatory remodeling and chronic pathological inflammation (Moustakli et al., 2026; Varga et al., 2025; Accogli et al., 2023; Yao et al., 2026). Therefore, rather than functioning as an indispensable molecular switch, NLRP3 may represent one component of a broader regulatory network that integrates inflammatory, metabolic, and redox signals.

In this hypothesis article, we propose a redox-inflammasome threshold model of ovulatory regulation, in which NLRP3 inflammasome signaling may serve as an important integrator of cellular stress signals that contributes to establishing the inflammatory threshold required for efficient follicular remodeling. According to this conceptual framework, successful ovulation may depend on the balance between adequate inflammasome activation to support tissue remodeling and excessive or sustained activation that promotes ovarian dysfunction. This model provides a new perspective for understanding the interaction between redox metabolism, innate immunity, and reproductive physiology, while generating experimentally testable hypotheses regarding the roles of NLRP3 signaling in disorders such as PCOS, POI, and ovarian aging.

## 2 NLRP3 inflammasome: a redox-sensitive integrative component

NLRP3 inflammasome is a redox-sensitive innate immune sensor that may integrate oxidative stress, metabolic danger signals, and inflammatory cues during ovarian remodeling (Figure 1) (Xu and Núñez, 2023; Zhang and Wang, 2020; Shi et al., 2020; Fu and Wu, 2023). Although inflammasome-related signaling has been implicated in ovarian physiology and pathology, direct evidence establishing NLRP3 as a determinant of physiological follicular rupture remains limited. We therefore consider NLRP3 here as a proposed integrative component of the ovulatory inflammatory network rather than as an indispensable regulator. The following framework summarizes potential links between hormonal priming, redox/metabolic stress, inflammasome activation, and periovulatory tissue remodeling.

**FIGURE 1:**
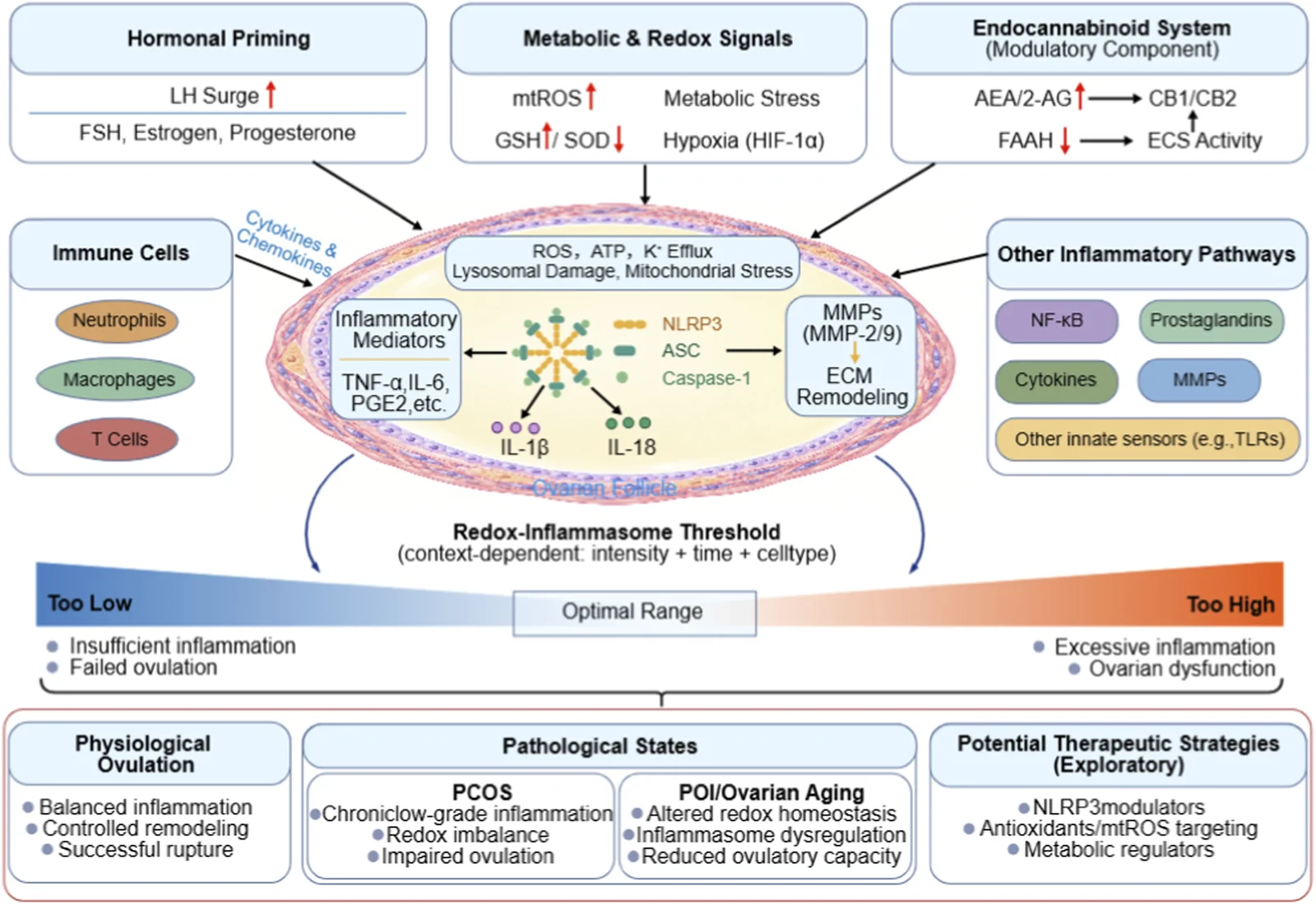
Proposed redox-inflammasome threshold model of ovulatory regulation. This conceptual framework illustrates the hypothesis that hormonal signals, redox and metabolic cues, cellular stress, and multiple inflammatory pathways may converge to shape periovulatory tissue remodeling. NLRP3 inflammasome signaling is presented as a modulatory component that may integrate cellular stress signals rather than as a necessary or sufficient determinant of follicular rupture. The proposed threshold is dynamic and context-dependent, with outcomes ranging from inadequate remodeling to physiological remodeling or excessive inflammatory activation. The model is hypothesis-generating and does not imply a quantitatively established threshold or a single dominant ovarian cell type. Created with BioGDP.com.

### 2.1 Priming: hormonal licensing of inflammasome readiness

NLRP3 inflammasome activation generally involves a priming phase followed by activation in response to cellular danger signals (Accogli et al., 2023). In the ovary, LH-associated signaling promotes inflammatory and tissue-remodeling programs, including NF-κB-related responses and expression of ovulatory mediators (Richards et al., 2002b; Kelley et al., 2019). We hypothesize that these signals may create a permissive state for subsequent inflammasome activation. However, the extent to which LH directly licenses NLRP3 activation in specific periovulatory cell populations remains to be established.

During ovulation, LH surge promotes steroidogenic and inflammatory signaling, including COX-2 and PGE2 pathways (Richards et al., 2002b). These observations provide a plausible hormonal context for inflammasome priming, but they do not establish that LH-induced NLRP3 expression is necessary for follicular rupture. Thus, in the present model, hormonal signaling is viewed as an upstream input that may modify the cellular readiness for inflammatory activation.

Once priming has occurred, subsequent redox, metabolic, and tissue-injury signals may provide activating inputs. In accordance with the canonical two-signal framework, these inputs could promote inflammasome assembly and cytokine maturation (Accogli et al., 2023; He et al., 2016). Whether this sequence occurs in the same ovarian cells during physiological ovulation, and whether it is required for follicular rupture, remains an important experimental question.

### 2.2 Activation: redox and metabolic danger signals

The activation phase represents a proposed convergence point for redox and metabolic danger signals. NLRP3 is responsive to mitochondrial dysfunction, ROS, extracellular ATP, ionic disturbances, and lysosomal stress in multiple biological contexts (Xu and Núñez, 2023; Zhang and Wang, 2020; Shi et al., 2020; Fu and Wu, 2023; Zhou et al., 2011; Swanson et al., 2019; Di Virgilio, 2007; Sharif et al., 2019; Zorina-Lichtenwalter et al., 2024; Hornung et al., 2008; Bulté et al., 2023; Wang et al., 2025a; Wang et al., 2025b). Some of these signals also occur during periovulatory remodeling (Shkolnik et al., 2011; Sorić et al., 2026; Zhang et al., 2012; Varga et al., 2025; Agarwal et al., 2005). We therefore hypothesize that their temporal and spatial convergence may modulate NLRP3 activity in the follicular microenvironment, while recognizing that direct ovarian evidence for each individual activation mechanism remains incomplete.

Mitochondria-derived ROS (mtROS) are established NLRP3-activating signals in several cellular systems (Fu and Wu, 2023; Zhou et al., 2011). Periovulatory follicles also exhibit dynamic changes in ROS and mitochondrial function (Shkolnik et al., 2011; Varga et al., 2025). These observations support the plausibility that mtROS may contribute to inflammasome signaling during ovulation; however, a specific mtROS concentration required for follicular rupture has not been established.

K^+^ efflux is a canonical NLRP3 activation mechanism, often linked to extracellular ATP and P2X7 signaling (Swanson et al., 2019; Di Virgilio, 2007; Sharif et al., 2019; Zorina-Lichtenwalter et al., 2024). ATP release and tissue injury may occur during periovulatory remodeling, providing a potential route by which extracellular danger signals could influence inflammasome activity. Nevertheless, direct evidence demonstrating that P2X7-dependent K^+^ efflux is required for physiological follicular rupture is currently lacking.

Lysosomal destabilization and cathepsin release can activate NLRP3 in several inflammatory contexts (Hornung et al., 2008; Bulté et al., 2023; Wang et al., 2025a; Wang et al., 2025b). Because periovulatory tissue remodeling involves protease activity, ECM degradation, and cellular turnover, lysosomal stress is considered here as a plausible but currently unconfirmed contributor to NLRP3 activation in the ovulatory follicle.

### 2.3 Execution: inflammasome-mediated follicular rupture

If NLRP3 inflammasome activation occurs during periovulatory remodeling, assembly of NLRP3-ASC-caspase-1 complexes could promote maturation of IL-1β and IL-18, as established in other inflammatory settings (Xu and Núñez, 2023; Zhang and Wang, 2020; Shi et al., 2020; Fu and Wu, 2023). IL-1β has documented roles in ovarian inflammatory signaling and can influence MMP expression and prostaglandin pathways (Takahashi et al., 2006; Vande Walle and Lamkanfi, 2024; Zhang et al., 2019). We therefore hypothesize that NLRP3-dependent cytokine maturation may contribute to, but is not necessarily required for, the inflammatory remodeling associated with follicular rupture.

IL-1β can promote MMP-2/MMP-9 expression and interact with COX-2/PGE2-associated inflammatory pathways (Curry and Osteen, 2003; Takahashi et al., 2006). These observations establish a biologically plausible connection between inflammatory cytokines and ECM remodeling, but they do not demonstrate that NLRP3 is upstream of these events or that NLRP3 activation is necessary or sufficient for follicular rupture. GSDMD-mediated pyroptosis is another possible downstream consequence of inflammasome activation (Chen and Smith, 2023), although its physiological relevance to normal ovulation remains to be determined.

## 3 The ovulatory threshold model

Ovulation is increasingly understood as a coordinated process involving endocrine signaling, inflammatory remodeling, redox adaptation, and metabolic changes (Duffy et al., 2019; Moustakli et al., 2026; Shkolnik et al., 2011; Sorić et al., 2026; Zhang et al., 2012; Varga et al., 2025; Agarwal et al., 2005). Building on these established observations, we propose that NLRP3 may function as one integrative component within this network rather than as a non-redundant gatekeeper. The proposed threshold model describes how multiple inputs could collectively influence the magnitude, timing, and cellular context of inflammatory activation.

### 3.1 Hormonal input

LH surge may provide an upstream hormonal input that establishes a permissive inflammatory state through pathways involving cAMP/PKA, NF-κB, COX-2, and PGE2 (Richards et al., 2002b; Kelley et al., 2019). In the proposed model, these signals may influence the readiness of periovulatory cells to respond to subsequent redox and metabolic cues. The model does not assume that LH intensity or PGE2 levels alone determine an inflammasome threshold.

### 3.2 Metabolic state

Redox and metabolic state may constitute additional inputs into the proposed threshold. Periovulatory increases in ROS, ATP availability, mitochondrial remodeling, and cellular stress provide biologically plausible signals capable of modulating NLRP3 in other contexts (Shkolnik et al., 2011; Sorić et al., 2026; Zhang et al., 2012; Varga et al., 2025; Agarwal et al., 2005; Xu and Núñez, 2023; Zhang and Wang, 2020; Shi et al., 2020; Fu and Wu, 2023; Zhou et al., 2011; Swanson et al., 2019; Di Virgilio, 2007; Sharif et al., 2019; Zorina-Lichtenwalter et al., 2024). We hypothesize that their integrated dynamics, rather than any single metabolite or ROS species, may influence the inflammatory state of the follicular microenvironment.

### 3.3 Cellular stress

Cellular stress may further modulate the proposed inflammatory threshold through mitochondrial dysfunction, mtDNA release, lysosomal stress, and protease activity (Zhou et al., 2011; Hornung et al., 2008; Bulté et al., 2023; Wang et al., 2025a; Wang et al., 2025b; Vande Walle and Lamkanfi, 2024; Zhang et al., 2019; Chen and Smith, 2023; Zhan et al., 2023). These mechanisms are well documented in inflammasome biology, but their relative contribution to physiological ovulation remains uncertain. Accordingly, the model treats cellular stress as a modulatory input rather than a demonstrated upstream requirement for NLRP3 activation during follicular rupture.

### 3.4 Operational definition and biological significance

Operationally, we define the proposed inflammatory threshold as a context-dependent state emerging from the combined magnitude and duration of inflammasome-related signals and downstream outputs, rather than as a single molecular concentration. Candidate readouts include NLRP3/ASC assembly, caspase-1 activity, mature IL-1β/IL-18, and GSDMD cleavage, together with mtROS and related redox measures. The threshold is therefore hypothesized to be both temporal and quantitative and may differ among granulosa cells, theca cells, stromal cells, and infiltrating immune cells. A permissive inflammatory state could facilitate tissue remodeling, whereas insufficient or excessive/sustained activation could be associated with impaired remodeling or tissue injury. These alternatives are testable rather than established predictions of the model.

## 4 Integration with hypoxia and metabolic reprogramming

Ovulation involves dynamic interactions among oxygen availability, metabolism, redox signaling, and inflammation. In this hypothesis, hypoxia-related signaling and metabolic reprogramming may provide additional context that modifies NLRP3 responsiveness. Because several mechanistic links discussed below are derived from non-ovarian inflammatory systems, they are presented explicitly as extrapolations that require validation in periovulatory follicles.

### 4.1 Hypoxia signaling and HIF-1α

HIF-1α coordinates hypoxic adaptation and metabolic responses and can interact with inflammatory pathways in multiple biological contexts (Semenza, 2012; Yadav et al., 2019; Tannahill et al., 2013). Evidence from non-ovarian systems suggests that HIF-1α may enhance glycolytic activity, influence mtROS generation, and interact bidirectionally with IL-1β/NLRP3 signaling (Yao et al., 2026; Tannahill et al., 2013). Based on these observations, we hypothesize that HIF-1α could modulate NLRP3 responsiveness during ovulatory remodeling. However, direct evidence that HIF-1α amplifies NLRP3 activation as a physiological event in ovulation is currently insufficient.

A bidirectional HIF-1α-inflammatory relationship has been described in several inflammatory and metabolic settings (Yao et al., 2026; Tannahill et al., 2013). In the present framework, IL-1β-dependent stabilization of HIF-1α and HIF-1α-dependent changes in inflammatory signaling are proposed as a potential amplification loop. This should be regarded as a mechanistic hypothesis rather than an established ovarian pathway.

### 4.2 Immunometabolism in granulosa cells

Enhanced glycolytic activity during granulosa-cell differentiation and ovulatory remodeling can alter the availability of metabolic intermediates (Chen et al., 2025). Succinate is a well-characterized immunometabolic signal that can inhibit prolyl hydroxylases and stabilize HIF-1α in inflammatory contexts (O'Neill and Pearce, 2016; Mills et al., 2016). Succinate-dependent NLRP3 activation through mtROS has also been described outside the ovary (Mills et al., 2016). We therefore propose that succinate-HIF-1α-redox signaling may represent a candidate metabolic route influencing inflammasome responsiveness during ovulation, but direct evidence linking this pathway to physiological follicular rupture remains to be established.

Granulosa cells also undergo mitochondrial remodeling during the periovulatory period (Wang X. et al., 2025). Mitochondrial dynamics, electron-transport activity, and membrane-potential changes may alter local redox signaling. NLRP3 can associate with mitochondria-associated membranes (MAMs) in inflammatory models (Zhou et al., 2011; Sun et al., 2025); however, the cited MAM evidence is not ovarian. Thus, MAM-dependent inflammasome assembly is considered a plausible mechanistic extrapolation that requires direct testing in periovulatory granulosa and other follicular cells.

### 4.3 Endocannabinoid system (ECS) coupling

The endocannabinoid system (ECS) is involved in ovarian physiology and has been linked to redox and inflammatory regulation (Battista et al., 2007; Maccarrone et al., 2015; Lin et al., 2026; Walker et al., 2019). Within the proposed framework, the ECS is considered a modulatory rather than obligatory component. Specifically, LH-associated changes in endocannabinoid signaling could potentially alter AEA/FAAH balance, mitochondrial function, and redox state, modifying the cellular context in which NLRP3 is activated. Evidence from non-ovarian systems indicates that CB1 signaling can affect mitochondrial function (Hebert-Chatelain et al., 2016) and that FAAH deficiency can alter inflammatory responses (Bátkai et al., 2007; Chiurchiù et al., 2018). Thus, the proposed sequence, LH/ECS modulation → mitochondrial/redox changes → altered NLRP3 responsiveness → inflammatory remodeling, is hypothesis-generating and requires direct ovarian validation.

Changes in AEA signaling have been associated with ROS and mitochondrial effects in reproductive and non-reproductive tissues (Walker et al., 2019; Hebert-Chatelain et al., 2016). These observations provide a possible link between ECS activity and redox status, but they do not establish that AEA-CB1 signaling is an upstream determinant of NLRP3 activation during ovulation.

Accordingly, the ECS-redox-NLRP3 relationship is best viewed as a candidate modulatory branch of the proposed threshold network. It may influence the amplitude or duration of inflammatory signaling without being required for follicular rupture. This distinction is important because the central model does not require every regulatory pathway to converge directly on NLRP3.

## 5 Pathophysiological implications

Ovarian disorders provide clinically relevant contexts in which redox and inflammatory regulation may become dysregulated. However, associations between NLRP3 activity and ovarian disease do not by themselves establish that NLRP3 controls physiological ovulation. The following sections therefore use PCOS, POI, and ovarian aging as contexts in which the proposed threshold framework may generate testable hypotheses rather than as direct validation of the model.

### 5.1 Polycystic ovary syndrome (PCOS)

PCOS is characterized by ovulatory dysfunction, metabolic abnormalities, chronic low-grade inflammation, and oxidative stress (Stener-Victorin et al., 2024). Increased ROS and NLRP3-related inflammatory markers have been reported in follicular fluid and granulosa cells from women with PCOS (Wang et al., 2023). These findings are consistent with a dysregulated redox-inflammasome environment. However, the proposed interpretation that PCOS represents an elevated ovulatory inflammatory threshold remains hypothetical and should be tested by directly comparing periovulatory inflammasome dynamics and functional rupture outcomes.

### 5.2 Primary ovarian insufficiency (POI)

POI is associated with oxidative stress, mitochondrial dysfunction, and inflammatory alterations (Chi et al., 2023; Wang et al., 2020). Experimental studies have implicated NLRP3/GSDMD-associated pyroptosis in granulosa-cell injury, supporting a potential role for inflammasome signaling in pathological follicular loss. Nevertheless, these findings concern ovarian dysfunction rather than direct evidence that a lowered NLRP3 threshold causes POI. The threshold interpretation is therefore presented as a hypothesis that may help organize existing observations.

### 5.3 Ovarian aging

Ovarian aging is accompanied by oxidative stress, mitochondrial dysfunction, and chronic inflammatory changes (Wang X. et al., 2025; Navarro-Pando et al., 2021). Notably, inhibition of NLRP3 has been reported to prevent aspects of ovarian aging and improve fertility-related outcomes in aged mice (Navarro-Pando et al., 2021). These findings argue against treating NLRP3 as an indispensable positive regulator of ovulation and instead support a context-dependent model in which excessive or sustained inflammasome activity may become detrimental. Whether transient NLRP3 activity contributes to physiological periovulatory remodeling remains unresolved.

## 6 Therapeutic targeting of the redox-inflammasome axis

Targeting the redox-inflammasome axis may provide useful experimental and, potentially, therapeutic opportunities, but current evidence does not establish that NLRP3-directed interventions restore physiological ovulation. NLRP3 inhibitors, antioxidants, and metabolic modulators may therefore be considered candidate strategies for testing whether redox–inflammasome signaling contributes causally to ovarian dysfunction (Vande Walle and Lamkanfi, 2024; Navarro-Pando et al., 2021; Zhang et al., 2023; Li et al., 2023; Shen et al., 2023). For example, MCC950 and related NLRP3 inhibitors provide pharmacological tools for probing inflammasome dependence, whereas N-acetylcysteine and other antioxidants can be used to test whether reducing oxidative stress alters inflammasome-related phenotypes (Walker et al., 2019; Chiurchiù et al., 2018; Wang et al., 2020). Metformin and AMPK-related interventions may additionally modify inflammatory and metabolic signaling (Shen et al., 2023). Importantly, therapeutic manipulation should account for the possibility that excessive suppression of physiological inflammatory remodeling could have unintended effects on ovulatory function.

## 7 Conclusion

In this hypothesis article, we propose a redox-inflammasome threshold model of ovulatory regulation, in which NLRP3 inflammasome signaling may serve as an important integrator of cellular stress signals that contributes to establishing the inflammatory threshold required for efficient follicular remodeling. According to this conceptual framework, successful ovulation may depend on the balance between adequate inflammasome activation to support tissue remodeling and excessive or sustained activation that promotes ovarian dysfunction. This model provides a new perspective for understanding the interaction between redox metabolism, innate immunity, and reproductive physiology, while generating experimentally testable hypotheses regarding the roles of NLRP3 signaling in disorders such as PCOS, POI, and ovarian aging.

The proposed threshold is expected to reflect the combined magnitude, duration, and cellular context of inflammatory signaling, with measurable outputs potentially including NLRP3/ASC assembly, caspase-1 activity, mature IL-1β/IL-18, GSDMD cleavage, and mtROS. Its physiological relevance may differ among granulosa, theca, stromal, and immune cells and may be shaped by alternative inflammatory pathways, including NF-κB, prostaglandin signaling, cytokine networks, and other innate immune sensors. These possibilities remain to be tested.

Importantly, the present model remains a hypothesis-generating framework rather than an experimentally validated mechanism. Future studies should combine conditional NLRP3 manipulation in defined ovarian cell populations with quantitative live-cell redox imaging, inflammasome readouts, single-cell and spatial transcriptomics, and direct measurements of follicular rupture and ovulatory efficiency. Such experiments could determine whether a reproducible redox-inflammatory threshold exists and whether NLRP3 is necessary, contributory, redundant, or dispensable under specific physiological or pathological conditions.

Overall, the redox-inflammasome threshold model provides a testable conceptual perspective linking endocrine, immune, metabolic, and redox signaling during ovulation. Its value lies not in establishing NLRP3 as a proven gatekeeper, but in generating experimentally tractable predictions about how inflammatory magnitude, timing, and cellular context may influence ovulatory remodeling and ovarian dysfunction.

## Data Availability

The datasets presented in this study can be found in online repositories. The names of the repository/repositories and accession number(s) can be found in the article/supplementary material.
